# Microsatellite Analysis of Museum Specimens Reveals Historical Differences in Genetic Diversity between Declining and More Stable *Bombus* Species

**DOI:** 10.1371/journal.pone.0127870

**Published:** 2015-06-10

**Authors:** Kevin Maebe, Ivan Meeus, Maarten Ganne, Thibaut De Meulemeester, Koos Biesmeijer, Guy Smagghe

**Affiliations:** 1 Department of Crop Protection, Faculty of Bioscience Engineering, Ghent University, Coupure links 653, Ghent, Belgium; 2 Naturalis Biodiversity Center, Darwinweg 2, Leiden, the Netherlands; University of Innsbruck, AUSTRIA

## Abstract

Worldwide most pollinators, e.g. bumblebees, are undergoing global declines. Loss of genetic diversity can play an essential role in these observed declines. In this paper, we investigated the level of genetic diversity of seven declining *Bombus* species and four more stable species with the use of microsatellite loci. Hereto we genotyped a unique collection of museum specimens. Specimens were collected between 1918 and 1926, in 6 provinces of the Netherlands which allowed us to make interspecific comparisons of genetic diversity. For the stable species *B*. *pascuorum*, we also selected populations from two additional time periods: 1949–1955 and 1975–1990. The genetic diversity and population structure in *B*. *pascuorum* remained constant over the three time periods. However, populations of declining bumblebee species showed a significantly lower genetic diversity than co-occurring stable species before their major declines. This historical difference indicates that the repeatedly observed reduced genetic diversity in recent populations of declining bumblebee species is not caused solely by the decline itself. The historically low genetic diversity in the declined species may be due to the fact that these species were already rare, making them more vulnerable to the major drivers of bumblebee decline.

## Introduction

All over the world different pollinator species are undergoing major declines (e.g. [1]). Generalist foragers like many bumblebees, that are essential pollinators in natural and managed ecosystems, are no exception to this general phenomenon [2–4]. Different hypotheses aim to explain the observed declines in bee populations, e.g. the impact of pathogen infections and pathogen spill-over from reared pollinators, the use of pesticides, diet specialization, landscape modification and loss of forage (as reviewed in: [1–5]). Although all these factors and their interactions influence pollinator populations at different locations and at different scales (from individual, to colony, to population), the agricultural intensification with increasing loss of habitats and forage resources, which started between 1950–1980 [4,6–8], is thought to be the key driver in Europe [5]. Genetic processes can also contribute to the observed decline as a consequence of, e.g. habitat fragmentation and limited migration. Indeed, low genetic diversity might threaten populations by limiting their ability to adapt to future environmental change [9–11]. For instant, it may predispose populations to disease epidemics [3,12]. Secondly, low diversity may result in inbreeding, thereby reducing individual fitness and threatening population extinction [10–11,13–14].

Based on contemporary specimens, several studies have shown that populations of declining bumblebee species have lower levels of genetic diversity compared to stable species [3,15–18]. However, as discussed by Lozier et al. [19], without information on the historic situation, the question remains: is this low diversity actually the result of recent declines, or is it due to historical, e.g. pre-decline, differences in genetic variation among species? Differentiating between these two causes could be achieved by comparison of the levels of genetic diversity of populations before the major drivers of bumblebee declines have acted [20–21]. Thus, by contrasting past and recent genetic diversity, one could obtain estimates of the magnitude of these drivers on population sizes and the levels of gene flow between these populations [17–18].

In this paper, we compared the genetic diversity of declining and more stable bumblebee species before their major recent decline (between 1950–1980) [6–8]. We used microsatellites to genotype a set of pin-mounted museum specimens of 4 stable bumblebee species: *Bombus pascuorum*, *B*. *hortorum*, *B*. *pratorum* and *B*. *lapidarius*, and 7 declining species: *B*. *muscorum*, *B*. *veteranus*, *B*. *ruderarius*, *B*. *sylvarum*, *B*. *humilis*, *B*. *ruderatus* and *B*. *subterraneus* [22]. Samples were all collected in the Netherlands (1918–1926) before the recent declines started (between 1950–1980) [4,6–8]. Furthermore, we compared our results with currently available data (time period: 1975–2009) on genetic diversity in bumblebees [16,23–29] to obtain further insights whether the genetic diversity is similar in historical and current populations of the same declining and stable species. Together, these findings contribute to our understanding of the changes in population genetic processes and can provide valuable information for the implementation of conservation strategies.

## Material and Methods

### Museum specimens and their distribution

**Fig 1:**
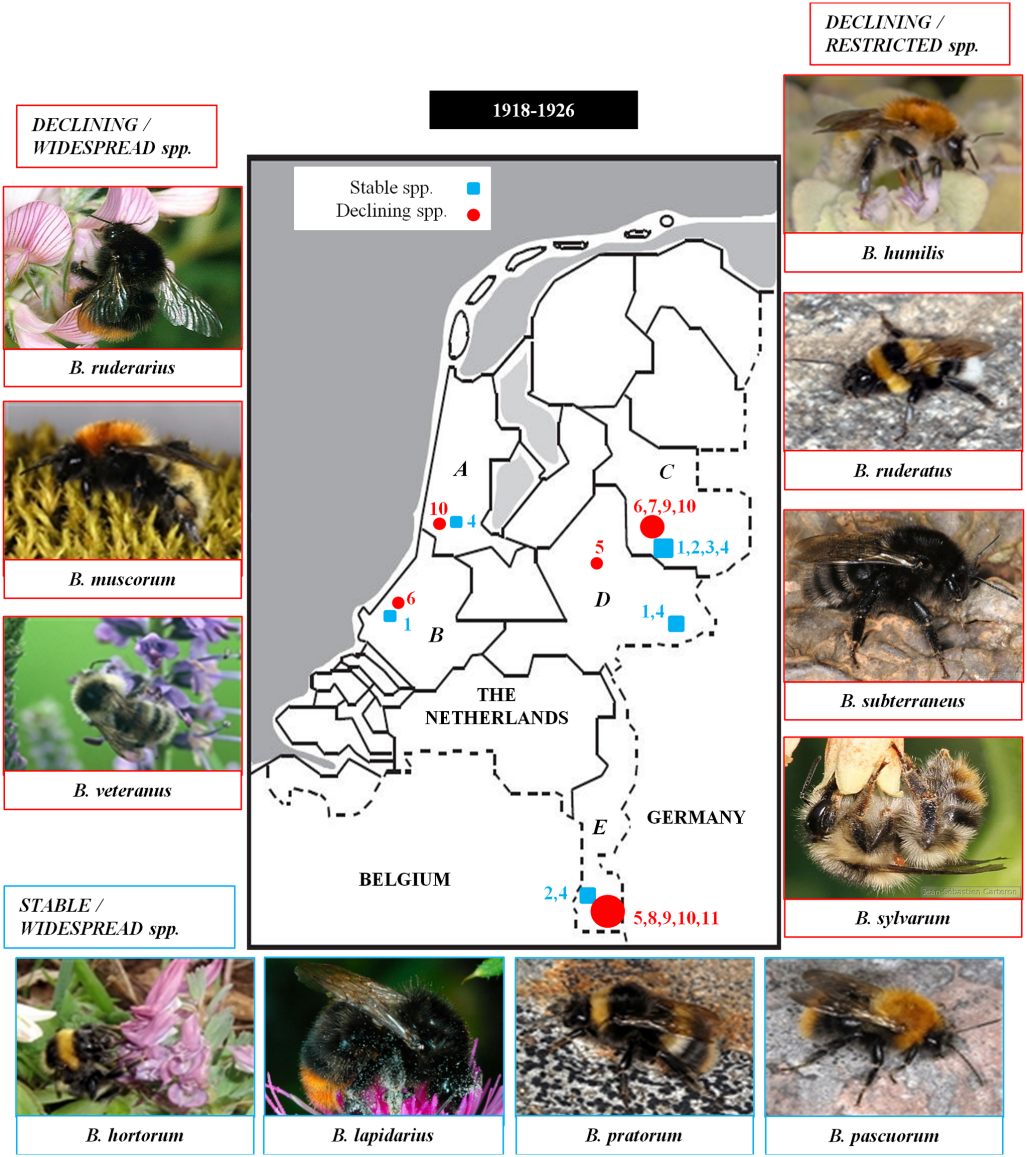
Distribution of the specimens of the declining and more stable *Bombus* spp. Specimens collected in the Netherlands between the years 1918–1926 before the recent bumblebee declines started (1950–1980), with a picture of each *Bombus* spp. used in the analysis. Species pictures from [56]. The letters refer to each sampling location: A = N-Holland, B = Z-Holland, C = Overrijssel, D = Gelderland and E = Limburg. Symbol size refers to the amount of species sampled at that location, while the numbers refer to which species. With for the stable and widespread species: 1 = *B*. *hortorum*, 2 = *B*. *lapidarius*, 3 = *B*. *pratorum*, 4 = *B*. *pascuorum*; for the declining and restricted species: 5 = *B*. *humilis*, 6 = *B*. *ruderatus*, 7 = *B*. *subterraneus*, 8 = *B*. *sylvarum*, and for the declining and widespread species: 9 = *B*. *muscorum*, 10 = *B*. *ruderarius*, and 11 = *B*. *veteranus*.

Museum specimens of 11 bumblebee species were selected from the Hymenoptera collection of the Naturalis Biodiversity Center in Leiden taking into consideration their distribution in the Netherlands (Fig 1).

We divided the selected species in groups based on their presence and status on the red list of the Netherlands [22] (S1 Table). Bumblebee species grouped as ‘declining’ have been given a red list status of ‘vulnerable’, ‘endangered’, ‘critically endangered’ or ‘disappeared’, while species grouped as ‘stable’ did not have a special red list status although these species also had range reductions. This first division of the species according to their red list status corresponds to the decline in their distribution (= trend, S1 Table). Here, species distribution is calculated as the relative areal size (i.e. the amount of hour blocks a species has been found / the total amount of hour blocks checked) x 100%, with an hour block representing a 5 x 5 km square area. The decline in distribution is calculated as: (the relative areal size of after 1970—relative areal size before 1970) / relative areal size before 1970) x 100% [22]. The species assigned to the ‘decline’ group showed a decline in distribution of 65% or more between 1970 and 2003, while for the ‘stable’ species the decline in distribution was less than 40% ([22], S1 Table). Furthermore, we divided the group of declining species in two based on their distribution before 1970: species with a distribution lower than 10% were considered as restricted (with mean (SD): 6.1% (2.8%)) while declining species with a distribution between 15–25% were considered as widespread (19.1% (2.4%); *T*-test, *t* = -6.465, d.f. = 5, *P* < 0.001). The group of declining and widespread species was not significantly different in range from the group of widespread but stable species (23.2% (2.8%); *T*-test, *t* = 1.937, d.f. = 4, *P* = 0.125).

Based on these criteria, we selected 4 stable and widespread bumblebee species: *B*. *pascuorum* (*N* = 38); *B*. *hortorum* (*N* = 30), *B*. *pratorum* (*N* = 10) and *B*. *lapidarius* (*N* = 19), 3 declining but widespread species: *B*. *muscorum* (*N* = 20), *B*. *veteranus* (*N* = 8) and *B*. *ruderarius* (*N* = 28), and 4 declining but restricted species: *B*. *sylvarum* (*N* = 16), *B*. *humilis* (*N* = 20), *B*. *ruderatus* (*N* = 17) and *B*. *subterraneus* (*N* = 7). Populations were collected in the period 1918–1926 and in 5 Dutch provinces: North-Holland, South-Holland, Gelderland, Overijssel and Limburg (Fig 1). Samples from a province were from one locality or different localities close together (within a 5 x 5 km frame). For all populations, 7 to 10 bumblebee workers were genotyped.

For *B*. *pascuorum*, we selected additional specimens from 4 populations of two more recent time periods, 1949–1955 (*N* = 40) and 1975–1990 (*N* = 49) and from one additional province: Drenthe. These extra 8 populations allowed us to investigate the genetic diversity of *B*. *pascuorum* over space and time. We suspected this to remain fairly stable as this species was and still is abundantly present in the Netherland. Deviations from this suspected result could indicate artefacts associated with the genotyping of museum specimens.

### DNA extraction and microsatellite protocol

Bumblebee DNA was extracted from one middle leg of each selected museum specimen with a Chelex method (InstaGene Matrix, BioRad) as described in [28]. Workers were genotyped at 10 microsatellite loci that have a size range lower or around 200 bp to avoid the chance of null alleles [20]: B11, B100, B121, B126 and B132 [30] and BT04, BT08, BT10, BT11 [31] as originally developed from *B*. *terrestris*, and BL02 [31] as derived from *B*. *lucorum*. Microsatellites were then amplified by PCR and visualized with capillary electrophoreses as described in [28]. Genotype replications of 48 random individuals were conducted to examine the genotypic error rate.

### Data analysis

Not all genotyped individuals of a population were included in the analysis due to several validation steps. First, specimens which could not be scored in a reliable manner for a minimum of 5 microsatellite loci, were excluded. Second, we used the program Colony 2.0 [32] employing corrections for genotyping errors (5% per locus) to search for the presence of multiple sisters from the same colony. To exclude problems using Colony 2.0 on populations with low genetic variability [33], we checked our data also with the program Kinalyzer [34] with both the ‘2 allele’ algorithm and the ‘consensus’ method. We randomly selected one individual per sibship for further analysis.

As the microsatellites used here were developed from *B*. *terrestris* and *B*. *lucorum*, we needed to validate if they could be used in a reliable manner in the different *Bombus* spp. We tested for genotypic linkage disequilibrium with FSTAT 2.9.3 [35] and for genotype frequencies against HW equilibrium expectations for each population of a species with GENALEX 6.3 [36]. When excess homozygosity was found, the program MICROCHECKER 2.2.3 [37] was used to check for evidence of null alleles.

### Genetic diversity

We estimated genetic diversity in each population using the allelic richness (*A*_*R*_) and Nei’s unbiased expected heterozygosity (*H*_E_; [38]). The latter statistic is not biased by the sample size of populations within the same species and appears not to be affected by null alleles [39]. The program HP-RARE [40], with hierarchical rarefaction to correct for sampling size, and GENALEX 6.3 [36] were used to estimate *A*_*R*_ and calculate *H*_E_ for each microsatellite locus, respectively. As some of our groups did not pass the Levine test, we used only nonparametric tests (e.g. Independent samples Mann-Whitney U test) in SPSS (version 21.0.0.0) to examine if the genetic diversity differed significantly between the widespread stable versus the restricted and widespread declining species and an ANOVA with Repeated Measures Factors to examine the genetic diversity between populations of *B*. *pascuorum*.

We conducted a sensitivity analysis of the calculated mean heterozygosity (*H*_E_) for each population of the different *Bombus* spp. in the time period 1918–1926 based on more stringent exclusion policies for missing data. We started this analysis from a maximum of 50% missing values (or 5 loci) within one specimen towards a more stringent exclusion step of only 10% (or one locus) missing data.

### Estimation of the genetic diversity and comparison between groups of species

Here, we used only a low number of specimens (n = 10 and after excluding step only 6–10) to estimate the genetic diversity for the different *Bombus* species. This is mainly due to the low number of available collection specimens and the study design of having a comparable sample size for all populations of each species. Normally, a reliable estimation of genetic diversity is made with 20–25 or more specimens of a population. Possible problems with low sampling size could be avoided by including more microsatellite loci. However, including more loci was not doable as DNA extracts were expended. Here, we still believe that our data is trustworthy as the estimations of genetic diversity for the different populations of a species are of a comparable magnitude and less then the differences in genetic diversity observed between the three different groups of species.

Furthermore, we compare the genetic diversity between groups of species. However, the interpretation of the observed inter-specific differences cannot be made easily due to: (i) mutation rates which may vary at different microsatellites loci and (ii) differences in polymorphism of the microsatellite loci. To remedy these effects, we used the same microsatellite loci for each species and bumblebee specimens with similar distribution in The Netherlands. In addition, we compared a group of 7 declining species with a group of 4 stable bumblebee species instead of single species. Furthermore, each group consisted of bumblebee species of multiple subgenera. In this way we minimize inconsistencies and perform a valid comparison between groups of species [17–19].

### Population structure

Genetic differentiation values (*F*_ST_) between the *B*. *pascuorum* populations within years and within populations between years were calculated using 1000 permutations in FSTAT 2.9.3 [35] and re-calculated after applying the ENA correction for null alleles as implemented in FREENA [41]. We also estimated the true measure of differentiation, Jost’s D [42], using the software SMOGD v2.6 [43].

## Results

### Data analysis

Genotype replications of random individuals showed only 4 specimens with an error at 1 of the 10 loci. Thus, we have a correct repetition of a single microsatellite locus of 99.2%.

Eight to ten microsatellite loci amplified in each *Bombus* species and were consistent across replicates (Table 1). Overall populations and species, we excluded in total 27 specimens which could not be scored in a reliable manner for a minimum of 5 microsatellite loci (Table 1). Analysis with Colony 2.0, controlled with Kinalyzer, revealed that almost all populations (28 out of the 31) contained 1 to 3 full-sib pairs (Table 1). We randomly selected one individual per sibship for further analysis. Of the in total 302 specimens, 234 specimens were kept for further analyses after removal of the 27 specimens with too many non-amplifications and removal of the 41 sisters (46 specimens of 4 restricted and declining species; 40 specimens of 3 widespread and declining species; and 148 specimens of the stable species; Table 1).

**Table 1:** Scoring efficiency of the microsatellite loci for each *Bombus* spp.

					PUA	PUA	PUA	PUA	PUA	PUA	PUA	PUA	PUA	PUA	
Group	*Species*	*n*	*NA*	*FS*	B11	B121	B132	B100	B126	BT11	BL02	BT04	BT08	BT10	*L*
**Widespread / Stable**	*B*. *hortorum*	30 (22)	3	5	0.0	0.0	25.0	0.0	31.3	0.0	70.0	0.0	18.8	0.0	10
	*B*. *lapidarius*	19 (12)	6	1	0.0	0.0	41.7	41.7	100.0	0.0	58.3	0.0	58.3	0.0	9
	*B*. *pratorum*	10 (8)	1	1	0.0	0.0	12.5	12.5	12.5	0.0	0.0	25.0	37.5	0.0	10
	*B*. *pascuorum*	127(106)*	6	15	2.8	0.0	10.4	100.0	1.9	3.8	100.0	14.2	3.8	0.0	8
**Restriced / Declining**	*B*. *humilis*	20 (16)	0	4	0.0	0.0	25.0	0.0	31.3	0.0	0.0	0.0	18.8	0.0	10
	*B*. *ruderatus*	17 (12)	2	3	100.0	0.0	41.7	0.0	0.0	0.0	58.3	41.7	16.7	0.0	9
	*B*. *subterraneus*	7 (7)	0	0	0.0	0.0	0.0	0.0	0.0	0.0	0.0	100.0	100.0	0.0	8
	*B*. *sylvarum*	16 (11)	3	2	9.1	0.0	54.5	0.0	54.5	0.0	0.0	0.0	45.5	0.0	10
**Widespread / Declining**	*B*. *muscorum*	20 (15)	3	2	6.7	6.7	46.7	6.7	6.7	0.0	0.0	0.0	33.3	0.0	10
	*B*. *ruderarius*	28 (18)	3	7	61.1	0.0	27.8	0.0	0.0	0.0	0.0	0.0	27.8	27.8	10
	*B*. *veteranus*	8 (7)	0	1	0.0	0.0	14.3	0.0	14.3	0.0	14.3	0.0	14.3	0.0	10
	**TOTAL**	302(234)*	**27**	**41**											**9.5**

With *n* = the number of workers and between brackets the number of workers used in all further analysis, *NA* = the number of specimens that were not amplifiable, *FS* = the number of full sibs, and *PUA* = the proportion of unsuccessfully amplified individuals per locus (in %). Microsatellite loci not used for further analysis are underlined with a full line, loci that were not used in only one population of a certain species are underlined with a dotted line, *L* = the number of loci used in further analysis.

* *=* workers of *B*. *pascuorum* from two additional time points: 1942–1960 and 1975–1995.

No significant linkage disequilibrium was found between the pairs of loci, when testing each locus pair across populations. All loci displayed heterozygote deficits under the Hardy-Weinberg equilibrium for at least one population of a species, which is indicative for the presence of null alleles. However, MICROCHECKER 2.2.3 revealed low (<10%) null allele frequencies for those loci.

### Genetic diversity and differentiation of *B*. *pascuorum*

**Fig 2:**
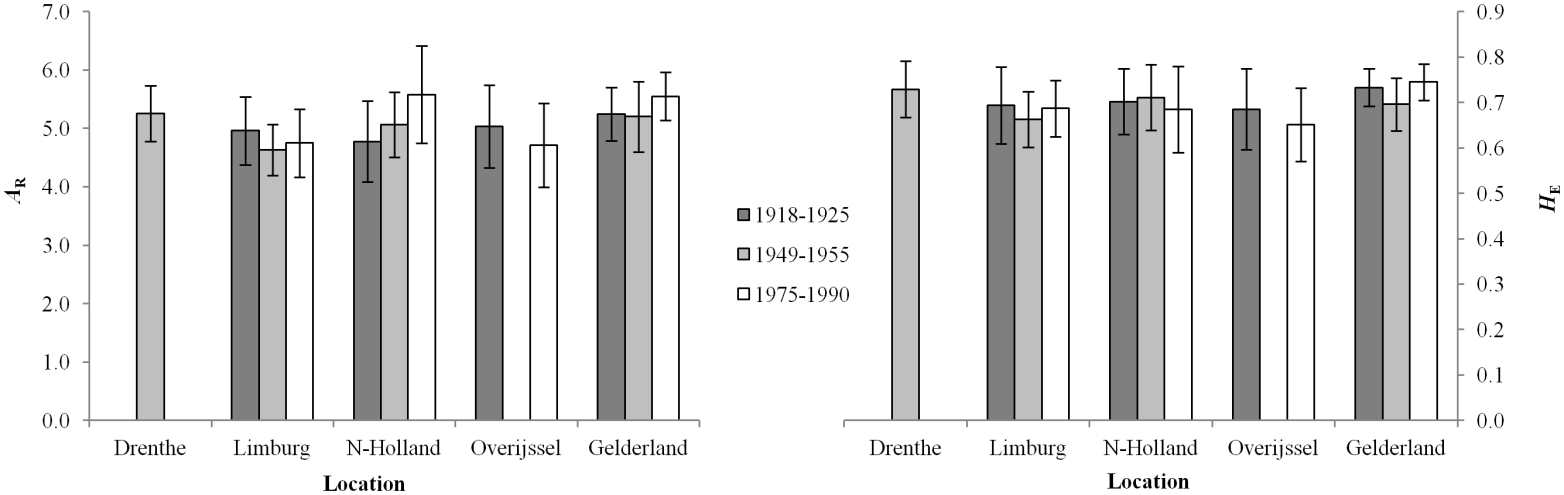
Genetic diversity of the *Bombus pascuorum* populations. The mean allelic richness (*A*_R_) and expected heterozygosity (*H*_E_) averaged across loci (and S.E.) between the *B*. *pascuorum* populations over the different locations and the three time periods.

Before analyzing all *Bombus* species, we estimated the genetic diversity of all *B*. *pascuorum* populations over the three time periods. As this species was and still is abundantly present in the Netherlands, we expected the genetic diversity to be fairly stable in space and time. If we detect a low genetic diversity in the past for *B*. *pascuorum*, this could suggest artefacts associated with the genotyping of museum specimens, such as the presence of null-alleles. Here, the genetic diversity of the *B*. *pascuorum* populations remained stable as there were no significant differences in genetic diversity over the different locations (ANOVA with Repeated Measures Factors; *A*_R_, *F* = 1.032, df = 4, *p* = 0.408; *H*_E_, *F* = 1.262, df = 4, *p* = 0.308) and the three time periods (ANOVA with Repeated Measures Factors, *A*_R_, *F* = 0.0116, df = 1, *p* = 0.743; and *H*_E_, *F* = 0.276, df = 1, *p* = 0.615; Fig 2). Thus, the microsatellite analysis of old specimens was reliable.

Comparison of the *B*. *pascuorum* populations within and between the different time periods revealed only in a few cases significant genetic differentiation (*F*_ST_) (S2 Table). Correction for the occurrence of null alleles, i.e. the ENA correction, had no effect on the genetic differentiation. Furthermore, the calculation of Jost *D*, another statistic to measure differentiation, within each time period was low: 0.057 for 1918–1926, 0.060 in 1949–1955, and 0.013 in 1975–1990, and not significantly different from zero (one sample T-test against 0, *t* = 2.202, *p* = 0.064; *t* = 1.742, *p* = 0.125; and *t* = 1.204, *p* = 0.268; respectively). So, *B*. *pascuorum* populations showed no or only marginal genetic differentiation.

### Genetic diversity in declining versus stable species

**Fig 3:**
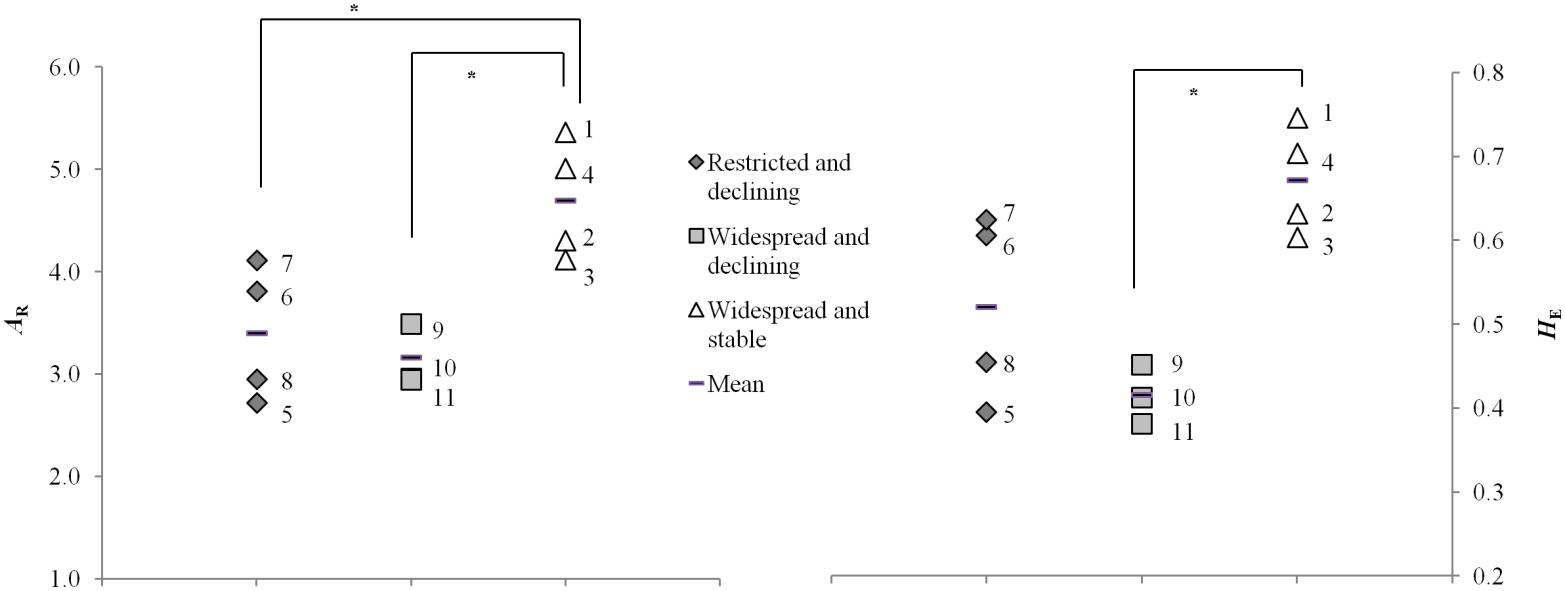
Historical genetic diversity of declining versus stable bumblebee species. Comparison of the mean allelic richness (*A*_R_) and expected heterozygosity (*H*_E_) averaged across loci between the populations of the declining and more stable *Bombus* species within the time period 1918–1926. With indication of the significance level, * = *P* < 0.05

For each population of the declining and stable species, we estimated the genetic diversity (Table 2). Next, we assessed whether declining *Bombus* species had a lower genetic diversity than stable species before their recent decline (Fig 3). The allelic richness (*A*_R_) and expected heterozygosity (*H*_E_) of the declining species: 3.218 (SE 0.158) and 0.466 (SE 0.030), were significantly lower than that of the stable bumblebee species with 4.883 (SE 0.164) and 0.692 (SE 0.022) (*A*_R_ and *H*_E_, respectively) (Mann-Whitney U test, *Z* = -2.646, *p* = 0.006; and *Z* = -2.268, *p* = 0.024; Table 2). Although two declining species (*B*. *ruderatus* and *B*. *subterraneus*) had a comparable mean *H*_E_ as some of the stable species (Fig 3).

**Table 2:** Historical genetic diversity within all *Bombus* species.

				*A*_R_		*H*_E_	
Species	Location	Year	*n*	Mean	SE	Mean	SE
**Widespread / stable**							
*B*. *hortorum*	Gelderland	1918	8	5.095	0.826	0.697	0.076
	Overijsssel	1918	7	5.490	0.465	0.763	0.034
	Z-Holland	1923	7	5.502	0.488	0.778	0.026
*B*. *lapidarius*	Limburg	1918	5	4.000	1.000	0.553	0.125
	Overijssel	1918	7	4.604	0.571	0.710	0.041
*B*. *pratorum*	Overijssel	1918	8	4.114	0.640	0.604	0.084
*B*. *pascuorum*	Limburg	1918	9	4.962	0.582	0.694	0.085
	N-Holland	1924	9	4.777	0.692	0.702	0.072
	Overijssel	1918	8	5.035	0.704	0.685	0.089
	Gelderland	1925	7	5.250	0.457	0.733	0.041
	**Total**		**75**	***4*.*883***	***0*.*164***	***0*.*692***	***0*.*022***
**Restricted / declining**							
*B*. *humilis*	Gelderland	1926	8	2.907	0.455	0.425	0.097
	Limburg	1918	8	2.527	0.415	0.366	0.094
*B*. *ruderatus*	Z-Holland	1923	5	3.571	0.649	0.543	0.103
	Overijssel	1918	7	4.044	0.300	0.669	0.031
*B*. *subterraneus*	Overijssel	1925	7	4.111	0.526	0.625	0.078
*B*. *sylvarum*	Limburg	1918	6	3.116	0.642	0.451	0.118
	Limburg	1920	5	2.778	0.547	0.458	0.101
	***Subtotal***		***46***	***3*.*293***	***0*.*236***	***0*.*598***	***0*.*042***
**Widespread / declining**							
*B*. *muscorum*	Limburg	1918	7	3.222	0.621	0.401	0.112
	Overijssel	1918	8	3.750	0.596	0.503	0.099
*B*. *ruderarius*	Limburg	1918	7	3.519	0.607	0.496	0.112
	N-Holland	1924	5	3.167	0.654	0.490	0.104
	Overijssel	1918	6	2.184	0.539	0.252	0.103
*B*. *veteranus*	Limburg	1918	7	2.942	0.574	0.382	0.109
	***Subtotal***		***40***	***3*.*131***	***0*.*222***	***0*.*421***	***0*.*040***
	**Total**		**86**	**3.218**	**0.158**	**0.466**	**0.030**

Here, we describe the mean values (and SE) of the allelic richness (*A*_R_), and the expected heterozygosity (*H*_E_) for each *Bombus* spp. over all microsatellite loci and populations within the time period 1918–1926. With *n*: the number of samples used for this analysis after removal of the identified sisters.

The lower genetic diversity within the declining species as reported here could be the result of the smaller distribution range of some species in the declining group. This was not the case. Indeed, when we divided the species declining before 1970s in restricted and widespread species [22], the result remained the same. The genetic diversity of the widespread/declining group was significantly lower than that of the stable species (*A*_R,_ *Z* = -2.121, *p* = 0.034; and *H*_E_, *Z* = -2.121, *p* = 0.034) and the restricted/declining group was also significantly lower than that of the stable species for *A*_R_ (*Z* = -2.309, *p* = 0.021) and showed the same trend for *H*_E_ (Z = -1.732, *p* = 0.083, Fig 3). Both groups of declining species were not different from each other (*A*_R,_ *Z* = -0.354, *p* = 0.857 and *H*_E_, *Z* = -1.414, *p* = 0.229; Table 2 and Fig 3). This indicates that historically declining species already had a lower genetic diversity than bumblebee species with stable populations.

As we arbitrary chose to exclude specimens of which showed non-amplifications for 6 loci or more, there are still specimens in our analyses with non-amplifications which could have an effect on our results. However; the sensitivity analysis of the calculated mean heterozygosity showed that *H*_E_ was stable over the different exclusion steps (from five non-amplications per specimens allowed in the analyses to only 1 non-amplification per specimens, see also S3 Table). Most importantly, the significant differences of *H*_E_ between stable and declining species remained.

Furthermore, within each bumblebee species, a few populations had non-amplifications for all their individuals for one microsatellite loci, which could have an impact on our estimate of genetic diversity. To exclude the presence of these effects, we removed the populations which had non-amplifications for a certain microsatellite loci and we re-analyzed the genetic diversity of each population with the same 8 microsatellites (B11, B121, B126, B132, BT04, BT08, BT10, and BT11). This analysis showed also no major impact of the non-amplifications on our dataset (S4 Table).

## Discussion

### Genetic diversity in declining versus stable species

Our results showed that historical populations of declining bumblebee species had a significantly lower genetic diversity than found within the historical populations of co-distributed more stable species (Fig 3). In studies with recent specimens, this lower genetic diversity in declining bumblebee species is sometimes explained as a reduction in genetic diversity in response to environmental drivers (e.g. [17–18]). However, this latter hypothesis was formulated without having any information on the genetic diversity present within the populations of these bumblebee species before their major declines [17–18]. Interestingly, our results were obtained with museum specimens of nine decades ago, that is two to three decades before the declines of most bumblebees started. As reported for Belgium [7–8] and for the Netherlands and Britain [6] and reviewed in [18], general drivers like the reduction in floral resources by agricultural intensification started around 1950. Thus here, the observed difference in genetic variation between declining and stable bumblebee species was not due to a recent reduction in genetic diversity but was already present in the years 1918–1926. This result is relevant for the interpretation of other studies which solely used recent specimens to assess genetic diversity [15–19].

### Genetic diversity and rarity

Our results showed that declining bumblebee species had lower levels of genetic diversity than stable species. One possible explanation for the lower genetic diversity of the declining species in the early 20^th^ century could be a lower abundance of these species in this time period. Indeed, small bumblebee populations can have a reduced genetic diversity as a result of higher genetic drift [11,13]. However, there are indications that rarity alone cannot totally explain the observed low genetic diversity of the declining species: (i) some declining species were present in the collection with a magnitude comparable to some of the stable species between the years 1900–1940. However, this method is not fully reliable as it has caveats, e.g. collector biases and preference for collecting rare species over common ones [20]. (ii) by referring to historical publications or expert judgement indicating a fairly common status. No historical information of the Netherlands is present but some of these declining species were reported as abundant in Belgium [44–45]. For example: *B*. *veteranus* (then called *B*. *equestris*) ranked with second lowest allelic richness (2.942) was described as “fairly common” in Belgium [44–45]. However, as both indications have their own drawbacks, rarity is still a valid explanation of the low genetic diversity observed in the declining species. There are also some other possible explanations of the low genetic diversity in the declining bumblebees: (i) having small effective population sizes could be an intrinsic characteristic of those species. If this would be the case it makes those species originally more vulnerable for the major drivers of bumblebee decline; (ii) the genetic diversity in the populations of the declining species could be altered due to habitat fragmentation or population isolation events before the dates used in this study (1918–1926). Therefore we could search for a genetic bottleneck. However, the use of bottleneck tests for haplodiploid species is somewhat dubious, as there are many violations of the model assumptions certainly when the power is low due to low samples size [46]. So, we cannot exclude that the declining species had undergone a historical decline before 1918–1926. Thus, for now is not known what the cause of the observed differences in genetic diversity is. Further research is needed to investigate the causes of the reduced genetic diversity in the declined bumblebee species and thus to be able to distinguish between historical and the more contemporary causes.

### Historical versus recent level of genetic variation

**Fig 4:**
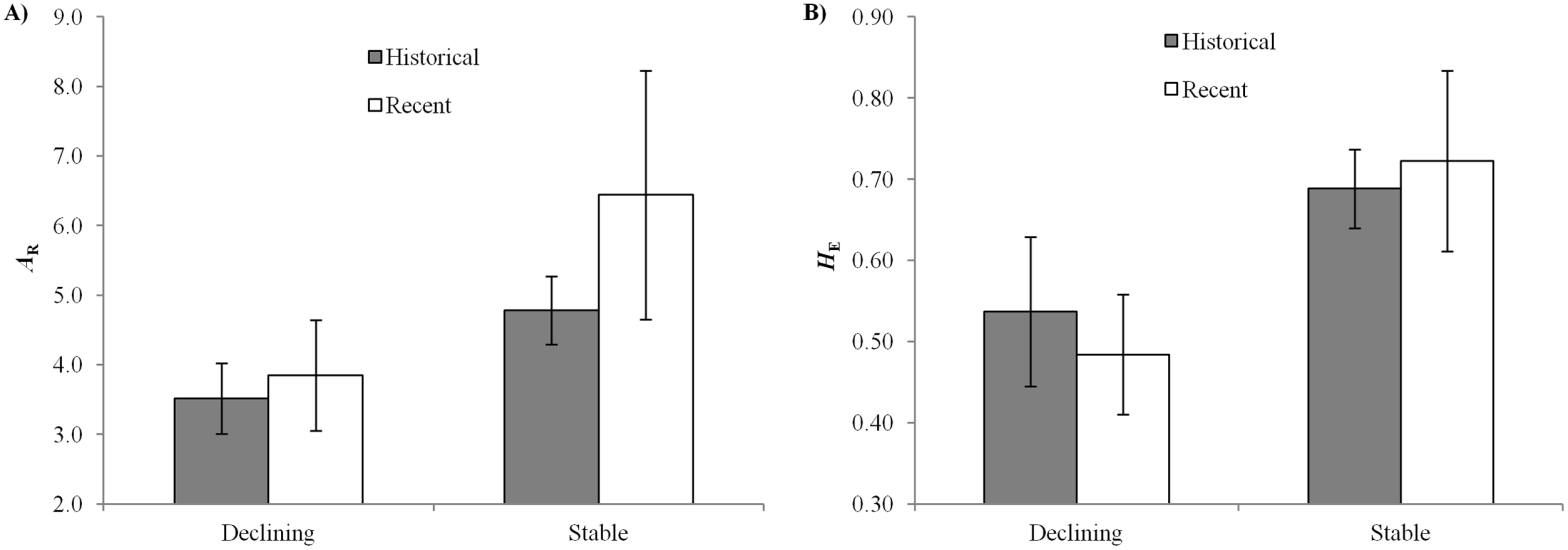
Historic versus recent genetic diversity of declining and stable bumblebee species. Comparison of the genetic diversity as the mean allelic richness (*A*_R_) and the expected heterozygosity (*H*_E_) averaged across loci (± S.D.) between the historical and recent data of a) the declining and b) the more stable bumblebee spp., with data from our project and from the literature [13,24–30]. See also S5 Table for referees and genetic parameters of these populations.

By comparing the levels of genetic variability among populations before a genetic bottleneck with those found in current populations, one will be able to determine if the observed lower genetic variation is a consequence of recent population declines, or an ancestral state [20–21]. Indeed, Bouzat et al. [47] showed a human-induced decrease in genetic diversity over time due to a decrease in allel number between historical and recent populations. However, other studies clearly showed that genetic diversity remains stable despite declines in population size [20,48–50]. We compared the genetic diversity of the declining and stable bumblebee species from our study also with the available data on genetic diversity from the literature (S5 Table). Although, we should compare the genetic diversity of historical populations with recent populations of the same species from the same location, this alternative comparison also indicates that the genetic diversity between historical and recent populations of stable and declining species remained fairly stable over the time (Fig 4). Only by contrasting past and recent genetic diversity, one could obtain estimates of the magnitude of the drivers on population sizes and the levels of gene flow between these populations. This could then be used for genetically monitoring populations for conservation and management [20,51]. Furthermore, as genetic variation is the raw material required for future adaptive potential, comparing genetic diversity levels between *Bombus* species may help to detect populations at risk of decline.

### Conservation

Our results have strong implications for conservation strategies. Determination of the genetic diversity of bumblebees can reveal which species are more vulnerable to local extinction in the longer term. Indeed, our study shows that all bumblebee species with a low genetic diversity and thus predicted to be vulnerable to decline, suffered more severe declines than the other species. However, it should be remarked that knowing the genetic diversity will not always identify which population is threatened. Indeed, two declining species showed similar levels of expected heterozygosity but had stronger declines than stable species with similar levels of heterozygosity (Fig 3). Thus, clearly also other factors than genetic diversity can play a role in the observed bumblebee declines. However and in general, these results suggest that determination of the genetic diversity is still a very good tool to predict bumblebee decline. Indeed, all five species with historically low genetic diversity levels (*H*_E_ lower than 0.550 and a *A*_R_ lower than 3.5) have subsequently suffered strong declines in their distribution. Hence, as bumblebee populations with high genetic diversity may be less likely to decline or to go locally extinct, improving the genetic diversity of the populations of restricted bumblebee species is a valuable strategy.

Being able to directly measure the genetic diversity from historical bumblebee populations allows for a better estimation of historical effective population sizes, levels of gene flow, and the relatedness between populations [20–21]. As the estimations of these population parameters are performed before the major (human) perturbations caused in the last decades, they are essential for making realistic conservation plans [20–21]. Furthermore, including historical data into conservation genetics could also help to put conservation goals into perspective [20–21]. Indeed, it could give an idea of a certain population size goal in the absence of recent scientific evidence. Such a clear framework of reference should be provide to avoid the dominance of more economic and politic reasons within conservation plans [21]. Indeed, several studies using historical specimens to estimate the effective population size showed that including historical samples can prevent us from reaching misleading conclusions [47,52–55].

In conclusion, our results demonstrate that species with a lower genetic diversity are the ones that are currently endangered. However, species with a high genetic diversity could still be at risk for extinction. Indeed, the more stable species also underwent distribution declines but not as severe as the declining group. So, to preserve bumblebee diversity one must tackle also the current drivers of bumblebee decline, to ensure that these low and even high genetic diversity species will not go extinct. It is therefore recommended that conservation strategies create more suitable habitat for sustaining bumblebee populations.

## Supporting Information

S1 Table Distribution, trend of decline and red list status of the different *Bombus* spp. In this table we presented, the distribution before and after 1970, trend of decline and red list status of the different *Bombus* spp. following Peeters and Reemer [1]. Species distribution is calculated as the relative areal size = (amount of hour blocks a species is found / the total amount of hour blocks checked) * 100%, with an hour block = 5 x 5 km square. The decline in distribution or trend is calculated by Peeters and Reemer [1] as: (the relative areal size of after 1970—relative areal size before 1970) / relative areal size before 1970 * 100%).
(PDF)

S2 Table Population structuring of the *B. pascuorum* populations. Pairwise *F*_ST_ (with ENA correction) for the different populations of *B*. *pascuorum* under the diagonal and the harmonic mean of *D*est across loci above the diagonal, a) between locations within a time period, and b) within a location between time periods. With indication of the significance level, ** = *P* < 0.001 and * = *P* < 0.005.
(PDF)

S3 Table Sensitivity analysis of genetic diversity. After removal of identified sisters, we conducted a sensitivity analysis of the calculated mean expected heterozygosity (*H*_E_) for each population of the different *Bombus* spp. in the time period 1918–1926, based on more stringent exclusion policies for missing data. From a maximum of 5 microsatellite loci with missing values within one specimen towards only one locus with missing data. With *n* = the total number of workers in each exclusion step and * *=* too low number of specimens.
(PDF)

S4 Table Estimation of genetic diversity after extra data exclusion steps. Recalculations of the genetic diversity were performed after removal of three species (*B*. *subterraneus*, *B*. *ruderatus* and *B*. *lapidarius*) and populations with non-amplifications and based on the same 8 microsatellite loci in each species.
(PDF)

S5 Table Comparison of the genetic diversity in historical and recent populations of declining and more stable bumblebee species. The data was obtained from our study and from the available data on recent populations found in the literature. With time periods: ‘historical’ = 1895–1930; and ‘recent ‘ = 1975–2010’.
(PDF)
