# Supplementary material for: Microsatellite Analysis of Museum Specimens Reveals Historical Differences in Genetic Diversity between Declining and More Stable Bombus Species
Source: PLoS One. 2015 Jun 10;10(6):e0127870. doi: 10.1371/journal.pone.0127870 (PMC4464549; doi:10.1371/journal.pone.0127870)
Supplement: S1 Table — In this table we presented, the distribution before and after 1970, trend of decline and red list status of the different Bombus spp. following Peeters and Reemer [1]. Species distribution is calculated as the relative areal size = (amount of hour blocks a species is found / the total amount of hour blocks checked) * 100%, with an hour block = 5 x 5 km square. The decline in distribution or trend is calculated by Peeters and Reemer [1] as: (the relative areal size of after 1970—relative areal size before 1970) / relative areal size before 1970 * 100%). (PDF) [file pone.0127870.s001.pdf]

S1_Table. Distribution, trend of decline and red list status of the different *Bombus* spp. In this table we presented, the distribution before and after 1970, trend of decline and red list status of the different *Bombus* spp. following Peeters and Reemer [1]. Species distribution is calculated as the relative areal size = (amount of hour blocks a species is found / the total amount of hour blocks checked) * 100%, with an hour block = 5 x 5 km square. The decline in distribution or trend is calculated by Peeters and Reemer [1] as: (the relative areal size of after 1970 - relative areal size before 1970) / relative areal size before 1970 * 100%).

	**Distribution in the Netherlands**					
	**Before 1970**		**1970-2001**			**Red list status**
**Species**	**Area size**	**Hour block**	**Area size**	**Hour block**	**Trend***	***1970-2003***
Widespread / stable						
B. pascuorum	42.2%	373	31.2%	343	-26.2%	
B. hortorum	20.3%	179	16.0%	176	-21.2%	
B. pratorum	23.5%	208	21.6%	238	-8.1%	
B. lapidarius	25.9%	229	16.1%	177	-37.9%	
Widespread / declining						
B. ruderarius	16.4%	145	5.5%	61	-66.2%	vulnerable
B. muscorum	21.0%	186	3.6%	40	-82.8%	endangered
B. veteranus	19.9%	176	1.2%	13	-94.1%	endangered
Restricted /declining						
B. humilis	7.8%	69	1.9%	21	-75.5%	endangered
B. sylvarum	5.3%	47	0.1%	1	-98.3%	critically endangered
B. ruderatus	8.7%	77	0.5%	6	-93.8%	critically endangered
B. subterraneus	2.5%	22	0.0%	0	-100.0%	Disappeared

1. Peeters TMJ, Reemer M (2003) Bedreigde en verdwenen bijen in Nederland (Apidae S.L.): Basis rapport met voorstel voor de Rode Lijst. Stichting European Invertebrate Survey, The Netherlands. (http://www.repository.naturalis.nl/document/46554)
