# Supplementary material for: Microsatellite Analysis of Museum Specimens Reveals Historical Differences in Genetic Diversity between Declining and More Stable Bombus Species
Source: PLoS One. 2015 Jun 10;10(6):e0127870. doi: 10.1371/journal.pone.0127870 (PMC4464549; doi:10.1371/journal.pone.0127870)
Supplement: S2 Table — Pairwise F ST (with ENA correction) for the different populations of B. pascuorum under the diagonal and the harmonic mean of Dest across loci above the diagonal, a) between locations within a time period, and b) within a location between time periods. With indication of the significance level, ** = P < 0.001 and * = P < 0.005. (PDF) [file pone.0127870.s002.pdf]

**S2_Table. Population structuring of the *B. pascuorum* populations.** Pairwise $F_{ST}$ (with ENA correction) for the different populations of *B. pascuorum* under the diagonal and the harmonic mean of *D*est across loci above the diagonal, a) between locations within a time period, and b) within a location between time periods. With indication of the significance level, ** = *P* < 0.001 and * = *P* < 0.005.

**a)**

| Limburg | 1918 | 1949 | 1989 |
|---|---|---|---|
| 1918 | - | 0.017 | 0.073 |
| 1949 | 0.018 | - | 0.079 |
| 1989 | 0.053** | 0.084* | - |

| N-Holland | 1924 | 1955 | 1980 |
|---|---|---|---|
| 1924 | - | 0.057 | 0.045 |
| 1955 | 0.012 | - | 0.001 |
| 1980 | 0.032 | -0.010 | - |

| Overijssel | 1918 | 1990 |
|---|---|---|
| 1918 | - | 0.032 |
| 1990 | 0.045 | - |

| Gelderland | 1925 | 1951 | 1975 |
|---|---|---|---|
| 1925 | - | 0.006 | 0.030 |
| 1951 | 0.002 | - | 0.000 |
| 1975 | -0.001 | 0.002 | - |

**b)**

| 1918-1925 | N-Holland | Limburg | Overijssel | Gelderland |
|---|---|---|---|---|
| N-Holland | - | 0.088 | 0.002 | 0.018 |
| Limbur | 0.039 | - | 0.012 | 0.059 |
| Overijssel | 0.023 | 0.011 | - | 0.008 |
| Gelderland | 0.013 | 0.040 | 0.032 | - |

| 1944-1955 | N-Holland | Limburg | Drenthe | Gelderland |
|---|---|---|---|---|
| N-Holland | - | 0.060 | 0.022 | 0.011 |
| Limburg | 0.036 | - | 0.058 | 0.057 |
| Drenthe | 0.070 | 0.050 | - | 0.068 |
| Gelderland | 0.022 | 0.075* | 0.033 | - |

| 1975-1990 | N-Holland | Limburg | Overijssel | Gelderland |
|---|---|---|---|---|
| N-Holland | - | 0.000 | 0.001 | -0.001 |
| Limburg | -0.009 | - | 0.042 | 0.003 |
| Overijssel | -0.014 | 0.012 | - | 0.014 |
| Gelderland | 0.013 | 0.001 | 0.004 | - |
