# Supplementary material for: Microsatellite Analysis of Museum Specimens Reveals Historical Differences in Genetic Diversity between Declining and More Stable Bombus Species
Source: PLoS One. 2015 Jun 10;10(6):e0127870. doi: 10.1371/journal.pone.0127870 (PMC4464549; doi:10.1371/journal.pone.0127870)
Supplement: S3 Table — After removal of identified sisters, we conducted a sensitivity analysis of the calculated mean expected heterozygosity (H E) for each population of the different Bombus spp. in the time period 1918–1926, based on more stringent exclusion policies for missing data. From a maximum of 5 microsatellite loci with missing values within one specimen towards only one locus with missing data. With n = the total number of workers in each exclusion step and * = too low number of specimens. (PDF) [file pone.0127870.s003.pdf]

S3_Table. Sensitivity analysis of genetic diversity. After removal of identified sisters, we conducted a sensitivity analysis of the calculated mean expected heterozygosity (H_E) for each population of the different *Bombus* spp. in the time period 1918-1926, based on more stringent exclusion policies for missing data. From a maximum of 5 microsatellite loci with missing values within one specimen towards only one locus with missing data. With *n* = the total number of workers in each exclusion step and * = too low number of specimens.

				Maximum microsatellite loci with missing values				
Species	**Location**	**Year**		***5***	**4**	**3**	**2**	**1**
			n	**161**	**159**	**154**	**139**	**112**
**Widespread / stable**								
B. hortorum	Gelderland	1918		0.697	0.697	0.708	0.688	- *
	Overijsssel	1918		0.763	0.763	0.763	0.767	0.767
	Z-Holland	1923		0.778	0.778	0.765	0.773	0.703
B. lapidarius	Limburg	1918		0.553	0.553	0.553	0.644	0.622
	Overijssel	1918		0.710	0.710	0.710	0.710	0.710
B. pratorum	Overijssel	1918		0.604	0.604	0.604	0.613	0.613
B. pascuorum	Limburg	1918		0.694	0.694	0.694	0.691	0.691
	N-Holland	1924		0.702	0.702	0.659	0.608	0.611
	Overijssel	1918		0.685	0.685	0.685	0.685	0.685
	Gelderland	1925		0.733	0.733	0.733	0.734	0.690
	**Total**			**0.692**	**0.692**	**0.687**	**0.691**	**0.677**
**Restricted / declining**								
B. humilis	Gelderland	1926		0.425	0.425	0.425	0.425	0.372
	Limburg	1918		0.366	0.366	0.366	0.366	0.299
B. ruderatus	Z-Holland	1923		0.543	0.543	0.543	0.509	0.493

Species	Location	Year	*Maximum microsatellite loci with missing values* 5	4	3	2	1
	Overijssel	1918	0.669	0.685	0.685	0.594	0.525
B. subterraneus	Overijssel	1925	0.625	0.625	0.625	0.625	0.605
B. sylvarum	Limburg	1918	0.451	0.451	0.451	0.470	0.484
	Limburg	1920	0.458	0.458	0.458	0.458	0.458
	Subtotal		***0.508***	***0.508***	***0.508***	***0.492***	***0.462***
**Widespread / declining**							
B. muscorum	Limburg	1918	0.401	0.383	0.383	0.383	0.389
	Overijssel	1918	0.503	0.498	0.498	0.498	0.498
B. ruderarius	Limburg	1918	0.496	0.496	0.496	0.570	0.570
	N-Holland	1924	0.490	0.490	0.490	0.496	0.458
	Overijssel	1918	0.252	0.252	0.252	0.250	0.289
B. veteranus	Limburg	1918	0.382	0.382	0.382	0.364	0.364
	Subtotal		***0.421***	***0.417***	***0.417***	***0.430***	***0.428***
	**Total**		**0.466**	**0.466**	**0.466**	**0.464**	**0.446**
