# Supplementary material for: Microsatellite Analysis of Museum Specimens Reveals Historical Differences in Genetic Diversity between Declining and More Stable Bombus Species
Source: PLoS One. 2015 Jun 10;10(6):e0127870. doi: 10.1371/journal.pone.0127870 (PMC4464549; doi:10.1371/journal.pone.0127870)
Supplement: S4 Table — Recalculations of the genetic diversity were performed after removal of three species (B. subterraneus, B. ruderatus and B. lapidarius) and populations with non-amplifications and based on the same 8 microsatellite loci in each species. (PDF) [file pone.0127870.s004.pdf]

S4_Table. Estimation of genetic diversity after extra data exclusion steps. Recalculations of the genetic diversity were performed after removal of three species (*B. subterraneus, B. ruderatus* and *B. lapidarius*) and populations with non-amplifications and based on the same 8 microsatellite loci in each species.

				A_R		H_E	
Species	**Location**	**Year**	***n***	**Mean**	**SE**	**Mean**	**SE**
**Widespread / stable**							
B. hortorum	Gelderland	1918	8	5.428	0.845	0.720	0.081
	Overijsssel	1918	7	5.515	0.429	0.779	0.025
	Z-Holland	1923	7	5.648	0.468	0.787	0.023
B. pratorum	Overijssel	1918	8	4.945	0.589	0.727	0.044
B. pascuorum	Limburg	1918	9	4.962	0.582	0.694	0.085
	N-Holland	1924	9	4.777	0.692	0.702	0.072
	Overijssel	1918	8	5.035	0.704	0.685	0.089
	Gelderland	1925	7	5.250	0.457	0.733	0.041
	**Total**		**63**	**5.195**	**0.118**	**0.728**	**0.014**
**Restricted / declining**							
B. humilis	Gelderland	1926	8	3.546	0.410	0.574	0.078
	Limburg	1918	8	3.182	0.363	0.522	0.072
B. sylvarum	Limburg	1918	6	3.821	0.610	0.601	0.089
	Limburg	1920	5	3.286	0.565	0.589	0.069
	Subtotal		***27***	***3.459***	***0.143***	***0.572***	***0.017***
**Widespread / declining**							
B. muscorum	Limburg	1918	7	3.603	0.640	0.516	0.109
	Overijssel	1918	8	4.360	0.517	0.613	0.078
B. ruderarius	Limburg	1918	7	4.149	0.594	0.620	0.102

				A_R		H_E	
Species	**Location**	**Year**	***n***	**Mean**	**SE**	**Mean**	**SE**
	N-Holland	1924	5	3.750	0.697	0.610	0.089
	Overijssel	1918	6	3.663	0.792	0.566	0.107
B. veteranus	Limburg	1918	7	4.153	0.423	0.619	0.064
	Subtotal		***40***	***3.946***	***0.128***	***0.591***	***0.017***
	**Total**		**67**	**3.751**	**0.144**	**0.583**	**0.014**
