# Supplementary material for: Microsatellite Analysis of Museum Specimens Reveals Historical Differences in Genetic Diversity between Declining and More Stable Bombus Species
Source: PLoS One. 2015 Jun 10;10(6):e0127870. doi: 10.1371/journal.pone.0127870 (PMC4464549; doi:10.1371/journal.pone.0127870)
Supplement: S5 Table — The data was obtained from our study and from the available data on recent populations found in the literature. With time periods: ‘historical’ = 1895–1930; and ‘recent ‘ = 1975–2010’. (PDF) [file pone.0127870.s005.pdf]

S5_Table. Comparison of the genetic diversity in historical and recent populations of declining and more stable bumblebee species. The data was obtained from our study and from the available data on recent populations found in the literature. With time periods: 'historical' = 1895-1930; and 'recent '= 1975-2010'.

***Bombus* species**	**Country**	**Sample size**	**Collection time**	H_E	**SE**	A_R	**SE**	**Reference**
**Declining *spp*.**								
B. humilis	Netherlands	16	1918-1926	0.396	0.096	2.717	0.435	This article
B. humilis	UK	150	2005	0.46	0.07	4.00	0.34	[22]
B. muscorum	Netherlands	15	1918-1926	0.477	0.107	3.514	0.620	This article
B. muscorum	UK	35.5	2003-2005	0.509	0.013	4.010	0.060	[21]
B. ruderarius	Netherlands	19	1918-1926	0.413	0.106	2.957	0.600	This article
B. ruderatus	Netherlands	12	1918-1926	0.606	0.067	3.808	0.475	This article
B. subterraneus	Netherlands	5	1918-1926	0.625	0.078	4.111	0.526	This article
B. sylvarum	Netherlands	11	1918-1926	0.455	0.110	2.947	0.595	This article
B. sylvarum	France	18	2004	0.53	0.09	4.00	0.85	[13]
B. sylvarum	UK	173	2003-2004	0.39	0.02	3.12	0.10	[13]
B. sylvarum	UK	150	2005	0.52	0.11	5.57	1.59	[22]
B. veteranus	Belgium	6	1895	0.607	0.062	3.470	0.345	[26]
B. veteranus	Belgium	34	1915	0.577	0.117	3.680	0.626	[26]
B. veteranus	Belgium	18	1923	0.578	0.118	3.710	0.645	[26]
B. veteranus	Netherlands	7	1918-1926	0.636	0.060	4.236	0.388	This article
**Stable *spp.***								
B. hortorum	Netherlands	22	1918-1926	0.746	0.045	5.362	0.593	This article
B. hortorum	UK	86	2003-2005	0.89		5.70		[23]
B. lapidarius	Netherlands	12	1918-1926	0.632	0.083	4.302	0.786	This article
B. pascuorum	Netherlands	33	1918-1926	0.704	0.036	5.013	0.292	This article
B. pascuorum	Netherlands	30.5	1975-1995	0.692	0.036	5.148	0.344	This article

***Bombus* species**	**Country**	**Sample size**	**Collection time**	$\boldsymbol{H_E}$	**SE**	$\boldsymbol{A_R}$	**SE**	**Reference**
B. pascuorum	UK	32	2003-2004	0.52	0.11	7.07	1.24	[13]
B. pratorum	Netherlands	8	1918-1926	0.671	0.057	4.460	0.603	This article
B. terrestris	Poland	238	2008-2009	0.720	0.072*	7.933	2.517*	[25]
B. terrestris	UK	24	1998-2000	0.826	0.019	5.079	0.700	[27]
B. terrestris	Spain	53	2003	0.600	0.080*	4.200	1.600*	[24]
B. terrestris	Germany	337	2004-2005	0.730	0.100*	7.150	2.200*	[24]
B. jonellus	UK	42	2003-2005	0.755	0.071	10.020	1.980	[21]

*** = SD used instead of SE**
